# 279 Structure and Leadership of Outpatient Antibiotic Stewardship Programs: Findings from the APIC MegaSurvey 2025

**DOI:** 10.1017/ash.2026.10640

**Published:** 2026-06-23

**Authors:** Tomislav Mestrovic, Ivayla Geneva, Dana Pepe, Bongyoung Kim, Rajalakshmi Ananthanarayanan, Maria Lourdes Bernadeth Manipon, JaHyun Kang, Sasheela Sri La Sri Ponnampalavanar, Vidya Mony, Keith H. St. John, Bassem Zayed

**Affiliations:** 1 University North; 2 Ivayla Geneva; 3 Beth Israel Deaconess Medical Center; 4 Hanyang University; 5 KIMSHEALTH; 6 National Kidney and Transplant Institute; 7 Seoul National University College of Nursing; 8 University Malaya Medical Centre; 9 Santa Clara Valley Healthcare; 10 Word Health Organization

## Abstract

**Background:** The Society for Healthcare Epidemiology of America (SHEA) International Ambassadors Program (IAP) aims to strengthen global infection prevention and healthcare epidemiology through engagement of emerging international leaders. Although program reach and activities have been described, factors associated with sustained engagement and post-program impact have not been quantitatively assessed. We conducted the first survey of current and former IAP participants to evaluate motivations, activities, barriers and factors associated with continued involvement. **Methods:** We administered an anonymous, cross-sectional online survey during 2023 to all identifiable current and former IAP members. The survey included 35 items capturing demographics, geographic region, areas of expertise, sources of program awareness, post-IAP activities, and ongoing engagement with SHEA. Descriptive statistics were calculated, followed by inferential analyses. Chi-square tests were used to assess associations between categorical variables, including geographic region and continued engagement with SHEA, as well as source of program awareness and participation in specific activities (training organization and conference presentations). One-way ANOVA was used to compare the mean number of reported activities across sources of program awareness. Statistical significance was set at p<0.05 (two-tailed). **Results:** Of 191 contacted ambassadors, 54 responded (27% response rate). Respondents were primarily based in Asia (50.9%), Latin America and the Caribbean (22.6%), and Africa (18.9%). Infection prevention and antimicrobial stewardship were the most common areas of expertise (51%), and nearly half learned about the program through former ambassadors. Over half (55.6%) reported ongoing engagement with SHEA through committees or other activities. No statistically significant associations were observed between geographic regions and continued SHEA engagement (p=0.065); also, no significant associations were observed between source of program awareness and organizing training programs (p=0.885) or conference presentation (p=0.897). Similarly, there was no significant difference in the mean number of reported activities by source of program awareness (ANOVA, p=0.621). Our findings indicate that post-program engagement and productivity were not dependent on recruitment pathway. Thematic analysis identified cost, time zone constraints, and language barriers as the most frequently reported obstacles to sustained participation. **Conclusion:** The SHEA IAP demonstrates meaningful global reach and impact on infection prevention activities. Ambassadors remain highly motivated and active, particularly in resource-limited settings, yet financial and logistical barriers limit long-term engagement. Addressing these challenges may enhance the program’s sustainability and global impact.

